# Amebic Liver Abscess

**DOI:** 10.4269/ajtmh.2010.10-0101

**Published:** 2010-11-05

**Authors:** Kanishka W. Garvin, James H. Willig

**Affiliations:** University of Washington, Seattle, Washington; University of Alabama at Birmingham, Birmingham, Alabama

**Figure 1.**
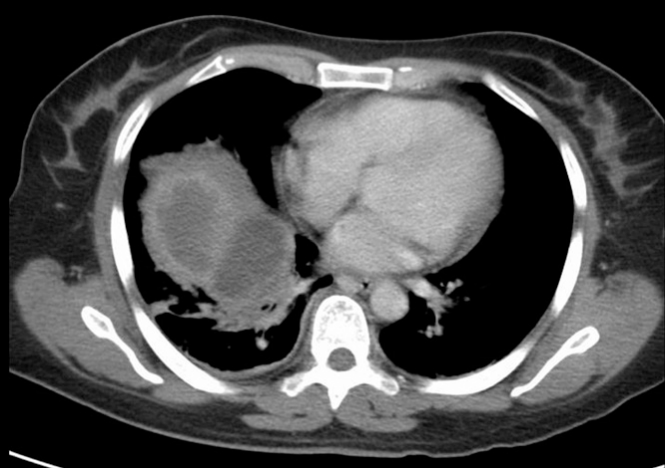
Initial abdominal CT scan demonstrating hepatic abscess.

**Figure 2.**
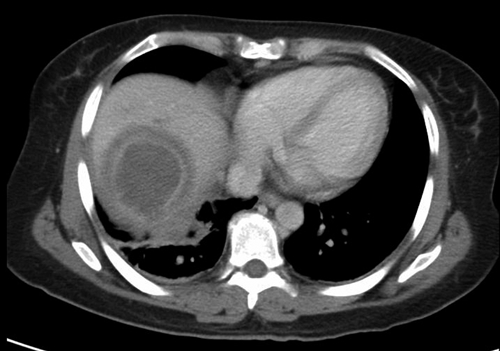
Initial abdominal CT scan demonstrating hepatic abscess.

A 45 year-old woman presented with a 6-week history of constant right upper quadrant abdominal pain, 20-lb weight loss, and worsening fevers. The patient had immigrated to the United States from Mexico 7 years earlier. She was febrile, experienced pain with light palpation of the right upper quadrant, and had normal serum transaminases, normal alkaline phosphatase, and mildly elevated total bilirubin of 1.1 mg/dL. Abdominal computerized tomography revealed contiguous peripherally enhancing, thick-walled, hypodense hepatic dome abscesses measuring 9.5 × 6.1 cm (Figures 1 and 2) *Entamoeba histolytica* serology was positive, and the size of the lesions halved after 4 days of metronidazole and symptoms rapidly resolved. *Entamoeba histolytica* is a protozoan that can cause asymptomatic colonization, amebic colitis, or extraintestinal abscesses.^1^–^3^ Transmission of infective cysts is fecal-oral, and patients usually have a history of exposure from an endemic area. Symptoms include a history of fever and right upper quadrant pain.^1^,^3^ Diagnosis can be aided with imaging studies and confirmed with serological studies and real-time polymerase chain reaction assays.^3^,^4^ Invasive disease should be treated with metronidazole for 10 days and paromomycin to eliminate colonization for 7 days.^3^ In rare cases, surgical intervention may be necessary.^3^
